# Facilitators, barriers and community perceptions for oral cancer prevention and control - A qualitative study from Madhya Pradesh, India

**DOI:** 10.6026/973206300212688

**Published:** 2025-08-31

**Authors:** Sana Anwar, Anindo Majumdar, Sanjeev Kumar, Pankaj Goel, Yogesh D. Sabde, Ujjawal Khurana

**Affiliations:** 1 Department of Community and Family Medicine, All India Institute of Medical Sciences, Bhopal, India

**Keywords:** Oral cancer screening, Madhya Pradesh, qualitative study

## Abstract

Oral cancer has high prevalence, morbidity and mortality, so it is of interest to document the barriers, facilitators and suggestions with respect to oral cancer prevention and control. Hence, total 42 participants were purposively selected from population for four focus group discussions, followed by thematic analysis of data summarized under three themes i.e, barriers, facilitators and suggestions. Key barriers were limited knowledge of oral cancer, personal behaviour, attitude and socio-cultural beliefs, suboptimal human resources, infrastructure, medicines and technology, transport issues. Facilitators were trust of population in ASHA workers and doctors, personal negative experiences and changing mindset of people. Suggestions included strengthening primary healthcare services, enhancing community engagement, using mobile technology for awareness, involving local leaders and empowering women.

## Background:

Oral cancer is a global health problem due to its high prevalence, incidence, morbidity and mortality [1]. As reported by GLOBOCAN 2020, more than 350,000 cases of oral cancer were diagnosed worldwide and approximately 180,000 people died from it each year [2]. In the majority of oral cancer cases, the disease is preceded by oral potentially malignant disorders (OPMDs) that can be clinically identified by healthcare professionals [3]. Early detection of these lesions is important, as prognosis becomes poorer significantly with delayed diagnosis [4]. The five-year survival rate for oral cancer is approximately 80% when diagnosed at stage 1 or 2, and around 20% when diagnosed at stage 3 or 4 [5]. Oral cancer has multiple etiology, with tobacco (smokeless and chewable) being the most important risk factor [6]. Alcohol, when taken along with tobacco has a significant synergetic effect [7]. Other risk factors include papillomavirus infections, a poor diet in fruits and vegetables, sunlight exposure, physical inactivity and heredity [8, 9]. In India, prevention of oral cancer, its screening, diagnosis and management comes under National Programme for Prevention and Control of Non-Communicable Diseases (NP-NCD) [10]. Previous studies have identified several barriers to early detection, including lack of awareness about the disease, misconceptions regarding its symptoms, and limited access to healthcare services, especially in rural areas [11]. Furthermore, socio-cultural factors, such as stigma and traditional beliefs, often influence health-seeking behaviours, resulting in delay or avoidance of medical treatment timely [12]. There is lack of robust qualitative studies from Madhya Pradesh regarding community perceptions and perceived barriers and facilitators related to oral cancer [13]. Understanding these perspectives that influence oral cancer related health behaviours is imperative for designing effective tailored interventions pertaining to the local context, aimed at prevention, care and control of oral cancer [14]. Qualitative research methods, such as focus group discussions (FGDs), helps to explore valuable information about the beliefs, attitudes, and behaviours of population/communities and also provides in-depth exploration of factors which often gets overlook when studies focus only on quantitative characterization of study population [15]. Therefore, it is of interest to report the barriers, facilitators and suggestions with respect to oral cancer prevention and control by exploring the views of the general population in two districts of Madhya Pradesh.

## Methodology:

The present study was a qualitative study using FGDs conducted between February and May 2024 in two districts of Madhya Pradesh namely Bhopal, primarily an urban population dominated district (named as district 1 in the present study) and Raisen, primarily a rural population dominated district (district 2). This study was part of a larger study aiming to find the effectiveness of an integrated public health intervention package for increasing oral cancer screening among adults and to find out the predictors of oral cancer screening. Participants were recruited from the general population, using purposive sampling as per various age groups, gender and residence. Total four FGDs were conducted, two in district 1, in the premises of primary health centre (PHC) and Ayushman Arogya Mandir respectively and two in district 2 in the premises of Anganwadis located in the area of a PHC. Four FGDs were conducted as data saturation was achieved. Participants were approached through ASHA workers of the selected area and a convenient time was mutually fixed. There were no dropouts and no participant refused to participate in the study. A topic guide was developed in local language based on literature review and expert inputs (co-authors). The topic guide included open-ended questions and prompts related to perceived barriers, facilitators and suggestions for prevention, care and control of oral cancer, including its risk factors, signs, symptoms, awareness and screening, and the same was pilot tested before the start of the study. All FGDs were facilitated face-to-face by the investigator (SA), female having BDS and MDS qualifications. She had received 3 days (10 hour) training in qualitative research methods and analysis from AM who is an experienced qualitative researcher. There was no prior relationship between SA and participants and neither had they known anything about the researcher. There was no personal bias or assumption from the interviewer's side. The interviews were recorded using a digital voice recorder. Field notes were taken by trained observer who was a local health functionary and covered a wide range of observations specific to the context. She was trained by SA. The audio recorded interviews were later transcribed verbatim into Hindi (local language) and then translated into English by SA.

The transcripts were not returned to participants for comments and/or correction due to lack of resources, time and refusal by some of the participants, and feedback was taken. Analysis was done through QSR Internationals NVivo 15 software. Both deductive and inductive approaches were used to analyse the data. Three main predefined themes were decided deductively namely barriers, facilitators and suggestions. For health system related information, six sub-themes were deductively pre decided based on World Health Organization's (WHO) health system building blocks framework i.e, service delivery, human resources, medicines and technology, health information systems, health financing, and leadership/governance. Simultaneously, inductive approach was also used to identify the other emerging sub themes that did not fit within the health system framework using thematic content analysis. These included coding and categorizing the data on knowledge, awareness, attitude, socio-cultural practices, personal behaviour, stigma, community engagement, NGOs and role of media. Inductive analysis was done through an iterative process involving reading and rereading of the transcripts. Data was coded by SA and checked and verified by AM. Codes with similar findings were clubbed into categories, similar categories were clubbed into sub-themes and finally themes were framed. Finally, all the authors confirmed and validated the themes and sub-themes. The study commenced after obtaining ethical approval from the Institutional Human Ethics Committee (IHEC-SR/PhD/July/22/01) and the COREQ guidelines are used for reporting [16].

## Results:

A total 42 participants were recruited from both the districts for the four FGDs. The demographic profile of the participants is given in table 1 (see PDF). The average duration of the FGDs was 43 minutes and 32 seconds. Three main themes were deductively framed namely- barriers, facilitators and suggestions whereas for sub-themes, some were structured deductively (for health system related data) and some inductively (non-health system related data) (Figure 1 and 2 - see PDF). Relevant verbatim quotes under each theme and sub-theme are mentioned in Table 2, 3, 4 (see PDF).

## Theme - barriers:

## Perceived lack of routine oral cancer screening and delays due to overburdened healthcare facilities:

Major barriers identified by the participants were perceived lack of routine oral checks ups, oral cancer screening, long queues, and delay in treatment and limited-service hours in their nearby public healthcare facilities. ASHA workers of some service areas were reportedly not carrying out their duties efficiently, and were focussed mainly on paper work and less on patient care. Delay in referrals to higher centres was also reported.

## Shortage of trained manpower and competing priorities:

Majority of the participants reported shortage of trained and specialized healthcare professionals such as dentists and oncologists. Irregular availability of doctors of PHC was also reported who according to the participants, had greater time engagement in meetings and field visits, due to which they could not give sufficient time to patients for explaining the relevant things clearly. Participants also reported lack of proper guidance from some of the staff of PHCs.

## Stock outs of medicines and unavailability of certain diagnostic equipment:

Issue of availability of diagnostic equipment and medicines at the PHC and sub-centre levels were also mentioned by the participants, including stock outs of medicines.

## Suboptimal delivery of health education and guidance regarding its use:

According to the participants, there was lack of community level awareness on oral cancer schemes, and there was no display of health education material like posters or public messages related to oral cancer awareness in the healthcare facilities, where they usually sought care from. The source of information stated was TV channels, radio, digital media or experiences shared by the patients or their relatives. Even though most people knew about the government scheme- Pradhan Mantri Jan Arogya Yojana (PMJAY) [17], which provides health insurance to needy families, only some had the Ayushman card required for claim, and also, there was confusion regarding how and where to use it.

## Lack of active involvement of community leaders in awareness generation and implementing public health measures:

Many participants reported that community leaders like sarpanch or panchayat members were not actively involved in spreading awareness about oral cancer. They highlighted that these village level functionaries approached them only during elections and were mostly unavailable for health-related issues. According to the participants, there were no checks on opening and free selling of gutkha and tobacco shops near schools and residential areas.

## Limited knowledge and awareness of causes and risk factors:

Many participants had limited knowledge about the full spectrum of causes of oral cancer and early recognition of symptoms. Some believed that the symptoms were due to spicy food or heat, so they did not visit the doctor.

## Transport issues:

Many participants shared that poor roads and lack or infrequent public transportation had made it even more difficult to reach hospitals, especially from remote areas. So, they had to rely on private vehicles. This according to them puts financial burden on poor families and often led to delay in seeking timely care.

## Personal behaviour, perceptions, attitude and socio-cultural beliefs:

Participants reported that social stigma, fear of diagnosis and socio-cultural beliefs were greatly responsible for delayed health seeking behaviour. People thought that having cancer is a matter of shame, and that, it is an incurable disease. Many had faith in traditional healers or local "babas". Women, in particular hesitated to speak or visit male doctors.

## Theme - facilitators:

Since some subthemes were similar to barriers, there were some mixed responses which meant that some participants reported some information within these subthemes as facilitators, whereas others reported them as barriers, especially with respect to service delivery, human resources and medicines and technology.

## Patient satisfaction with doctor's competency and behaviour:

Some participants reported satisfaction with the initial treatment, guidance and referrals given to them at their nearby public healthcare facilities. Doctors listened to them properly, were helpful in recognising the rare symptoms and referred them to higher centres for appropriate care.

## Human resources:

Good availability of doctors and supporting staff helped the participants to form a strong bond with them, as they appreciated the patience and time given to them. ASHAs guided them and helped them to reach to refer hospitals and even followed up on their health.

## Organisation of dental check-up camps and good availability of medicines:

Participants shared positive experiences about the organisation of dental camps and availability of mobile medical vans by NGOs and private healthcare facilities in their area for routine dental care, although these were not for screening. This helped particularly the elderly or those without transport to get checked. Good availability of essential medicines encouraged them to visit the doctor immediately in case of any health issues.

## Health financing:

Participants who used the Ayushman Bharat PMJAY scheme, found it useful for cancer treatment. This financial support helped the poor families to bear the treatment expenses. They didn't have to worry about arranging money for tests or medicines.

## Personal negative experiences:

Some participants reported that the bad experiences and the sufferings endured either by them or their family members due to addiction of tobacco products made them realize the harmful effects of these habits. Real life experiences motivated many to quit these habits, take the early symptoms seriously, and even made them advise others to stop using harmful products.

## Changing mindset of people:

A few people shared that their thinking has started to change and they are now more willing to go for mouth check-ups at regular intervals. Spread of awareness through mass media has reportedly shifted their mindset from traditional faith healers to modern treatment. They now realise the importance of early recognition for prevention and control of oral cancer.

## Theme suggestions:

## Addressing stigma and cultural norms:

Participants suggested a strong need to break the social stigma attached to oral cancer and change the cultural practices that prevent people from discussing about this disease. They also believed that women should be given more freedom to speak up about their health issues. Participants also advised that real life stories should be shared to make people realise the importance of early recognition of symptoms and harmful effects of treatment delay.

## Recruitment of trained health professionals:

Recruitment of more trained healthcare professionals such as dentists, cancer specialists, female doctors and supporting staff at the PHC and sub centre level was suggested. Suggestions also included training of ASHAs and ANMs for providing proper guidance on oral cancer care and health insurance schemes. Participants felt that people would understand better, if information was shared in simple language through ASHAs, mobile messages, or local announcements.

## Leadership and governance:

Participants suggested that involvement of community leaders like sarpanchs, ward members, and school principals in organizing awareness programs and health melas could be vital in spreading a strong message for oral cancer care. It was suggested that higher authorities should impose strict rules against the sale of gutkha and tobacco, especially near schools.

## Service delivery:

Participants also suggested that oral cancer screening camps should be held regularly in villages and slums, so that people don't have to travel far. They wanted their nearest government facility to offer oral health check-ups, diagnostic tests, imaging services, regular supply of medicines, and full time availability of staff, robust infrastructure and smooth referral system. They suggested that the government should explain the beneficiary schemes with greater clarity and guide people on how to utilize them more easily.

## Utilization of technology:

Participants suggested that mobile phones and digital platforms could be helpful in spreading awareness by sending voice messages, videos, and reminders about health camps and symptoms of mouth cancer. They also suggested the idea of using WhatsApp groups or community loudspeakers to reach more people. Mobile phones, WhatsApp, and recorded audio messages were reported as preferred sources for spreading awareness among masses. Telemedicine was also considered as good, provided someone assisted them in the process.

## Community based awareness:

Almost all participants felt that awareness must come from within the community. They suggested "nukkad natak [skits]", posters, mike announcements and health talks to be held at schools and anganwadis. People trust ASHA workers, school teachers, and the elderly, and so, involving them in awareness work was seen as effective. Some even said that they are ready to talk to neighbours, friends, and people at tea stalls to spread the message. Participants wanted awareness programs to be regular, creative, and focused on practical things like symptoms, harmful habits, and where to go for help.

## Discussion:

The present qualitative study explored the community perspectives on the barriers, facilitators, and suggestions related to oral cancer prevention and control in two districts of Madhya Pradesh. The findings of the study and the experiences shared by 42 people participating in four FGDs, highlighted the fact that challenges in oral cancer care are contributed by many factors working together such as people's habits, their beliefs, traditions and the healthcare system. A significant barrier identified in the present study was the lack of routine screening services and poor access to early diagnosis facilities. Similar barriers were reported in a study from rural Karnataka by Sankaranarayanan *et al.* where delayed referrals and lack of oral cancer awareness at the primary level effected early recognition [18]. This study also highlighted long waiting times and limited service hours at government healthcare facilities, which were consistent with the findings of Jadhav *et al.* from Rohtak district of Haryana, which also identified insufficient infrastructure and overburdened public health systems as barriers to timely care [19]. The present study reported poor availability of trained human resources, especially dentists and oncologists. This aligns with the findings of Shruti *et al.* who reported inadequate capacity for oral cancer screening in primary care settings in India [20]. The lack of female doctors was also an important finding in the present study, which affected women's health seeking behaviour. This is similar to the findings of systematic review on cancer screening uptake in low and middle income countries by Srinath *et al.* [21].

Financial constraints and insufficient information about Ayushman Bharat PMJAY scheme were reported in the present study. Similar findings of lack of clarity about PMJAY were also reported by Dixit *et al.* [22]. Transport related barriers mentioned in the present study were consistent with the study conducted by Mandengenda *et al.* in rural areas, where poor road infrastructure caused delayed treatment [23]. Findings of the present study showed that sociocultural factors, fear, stigma, and belief in traditional healers effected oral cancer care and prevention. This aligns with the findings of Brocklehurst *et al.* which emphasized the need for culturally appropriate health messaging in underserved populations [24] and Zhu *et al.* who found that stigma around oral cancer, influenced treatment decisions in postoperative patients in China [1]. In our study, these beliefs were particularly strong among women, which is similar to the findings reported by Goswami & Gupta. [25]. Despite these barriers, several facilitators also emerged in our study. Trust in ASHA workers and doctors, helped to build confidence in the public health system. This aligns with the findings of Hashim *et al.* where trust in healthcare providers improved uptake of cancer screening services [26]. Arrangement of mobile medical vans and camps by NGOs for other routine check-ups were appreciated in the present study and use of digital technology like WhatsApp and mobile reminders was suggested by the participants to spread awareness for oral cancer, which was similar to the findings from a recent digital intervention study in Gujarat conducted by Joshi *et al.* [27].

Real life experiences shared by oral cancer patients, either by themselves or through their families in our study encouraged the participants for behaviour and substance use change. This resonates with the study by Monteiro *et al.* which reported that personal storytelling and peer experiences are effective tools in cancer awareness programs [28]. Participants of our study suggested regular oral cancer screening camps, increased infrastructure and manpower at their nearest government healthcare facilities, restriction of tobacco products sales, training of healthcare workers and active participation by community leaders and representatives for oral cancer prevention and control. These are consistent with study conducted by Klingberg *et al.* on non-communicable disease control [29]. The strength of our study was that we explored the community perceptions on various aspects of oral cancer prevention and control in both urban and rural settings of Madhya Pradesh, using qualitative methods which helped to gain better understanding of this major public health concern in these districts, which did not have precisely reported comprehensive qualitative studies. One limitation of our study was that we could not get feedback on the transcripts from the community, which could have led to a possibility of researcher bias, which is a common issue in qualitative studies. Our study recommends the utilisation of community led awareness initiatives, strengthening primary care services, simplification and major efforts for greater understanding of government health schemes like Ayushman Bharat, greater utilization of technology, increasing trained manpower at the primary care levels, initiatives to decrease the social stigma associated with oral cancer and making women more empowered to speak-up regarding their health.

## Conclusion:

There is a need for multilevel comprehensive health system and non-health system interventions to address the barriers in oral cancer care. A multisectoral approach is essential for effective prevention, screening, diagnosis, treatment, and referral of oral cancer.

## Financial support and sponsorship:

Nil

## Declaration:

We hereby declare that no pre-print of this manuscript has been published elsewhere.
